# Supplementary material for: Silencing CDCA8 Suppresses Hepatocellular Carcinoma Growth and Stemness via Restoration of ATF3 Tumor Suppressor and Inactivation of AKT/β–Catenin Signaling
Source: Cancers (Basel). 2021 Mar 2;13(5):1055. doi: 10.3390/cancers13051055 (PMC7958635; doi:10.3390/cancers13051055)
Supplement: Supplementary file 1 [file cancers-13-01055-s001.pdf]

Supplementary Materials

Silencing CDCA8 Suppresses Hepatocellular Carcinoma Growth and Stemness via Restoration of ATF3 Tumor Suppressor and Inactivation of AKT/β–catenin Signaling

Taewon Jeon, Min Ji Ko, Yu-Ri Seo, Soo-Jung Jung, Daekwan Seo, So-Young Park, Keon Uk Park, Kwang Seok Kim, Mikyung Kim, Ji Hae Seo, In-Chul Park, Min-Ji Kim, Jae-Hoon Bae, Dae-Kyu Song, Chi Heum Cho, Jae-Ho Lee and Yun-Han Lee

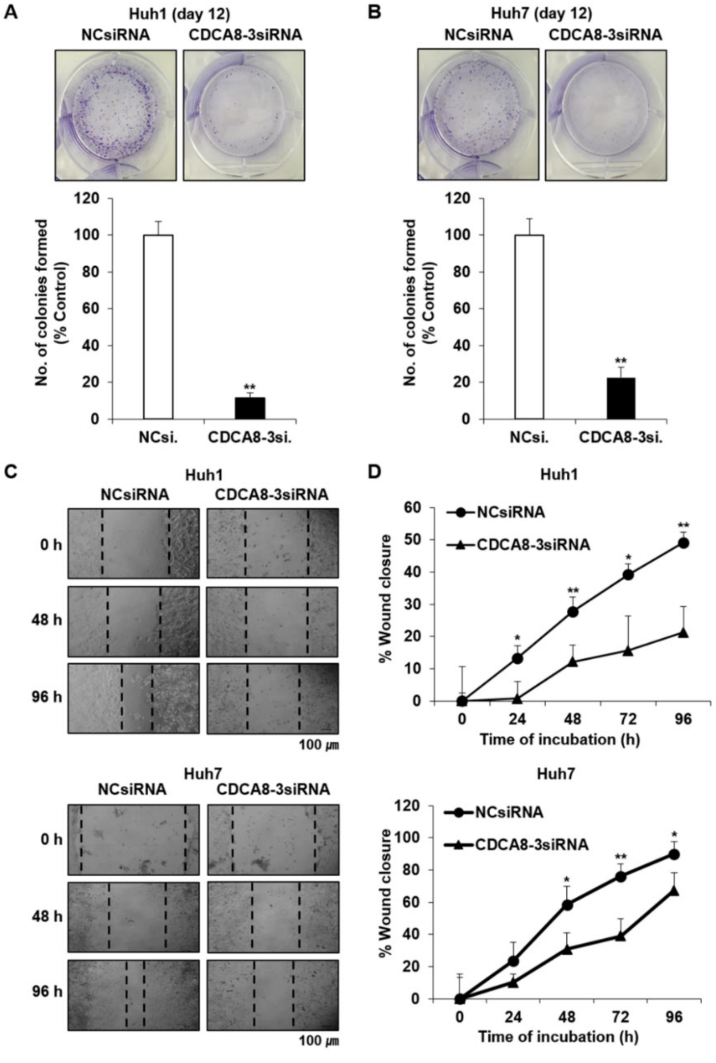

Figure S1. Treatment of CDCA8-3siRNA decreases clonogenicity and migration ability in HCC cells. (**A**,**B**) Observation of changes in the clonogenicity of Huh1 (**A**) or Huh7 (**B**) cells after 12 days of siRNA treatment. The number of colonies was also measured in each cell line (** $p < 0.01$ by the Poisson generalized linear model). (**C**,**D**) Observation of HCC cell migration using a wound healing assay. (**C**) Representative images were taken after 0 h, 48 h and 96 h of siRNA treatment. Scale bars, 100 μm. (**D**) Wound closure was measured daily for up to 4 days, and normalized to time point day 0 (* $p < 0.05$, ** $p < 0.01$ by Student's *t*-test).

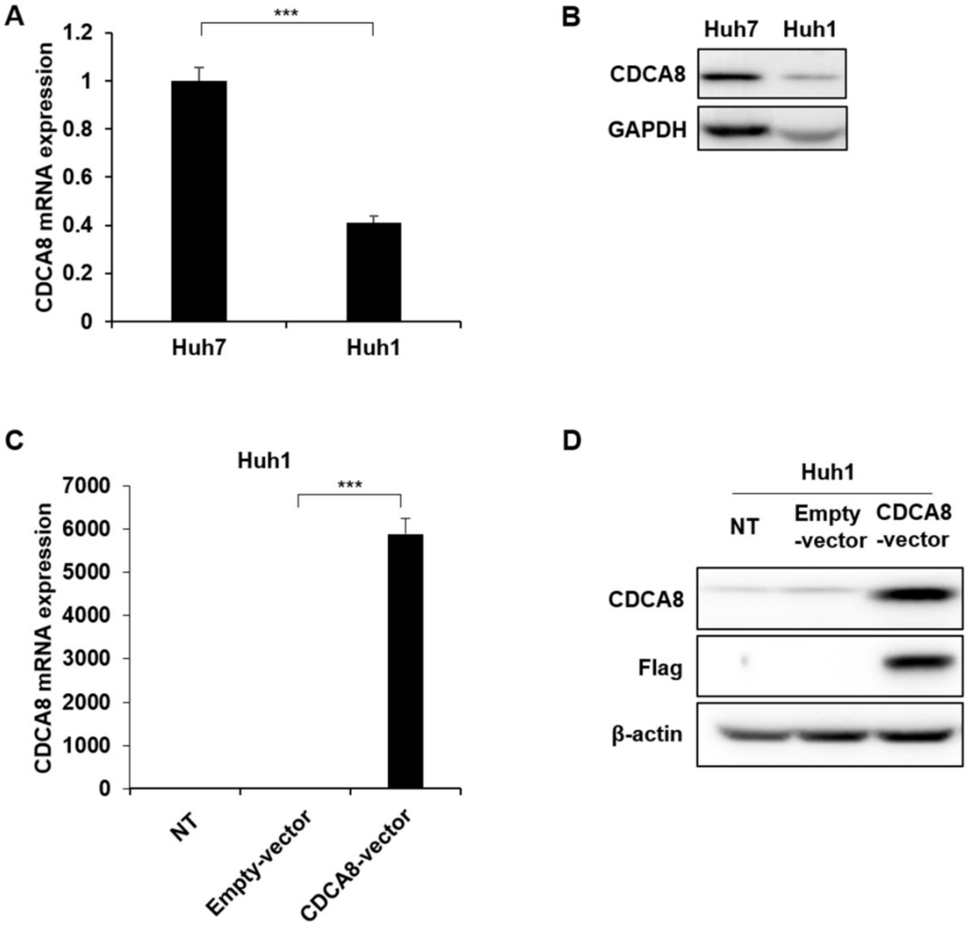

Figure S2. Basal expression of CDCA8 and induction of CDCA8 expression in HCC cells. (**A,B**) Relative basal levels of CDCA8 mRNA (**A**) or CDCA8 protein (**B**) in Huh1 and Huh7 cells. (**C,D**) Detection of CDCA8 mRNA (**C**) or CDCA8 protein (**D**) in Huh1 cells that were untreated (NT) or treated with control vector (Empty-vector) or CDCA8-expression vector (CDCA8-vector) (*** $p < 0.001$ by Student's t-test).

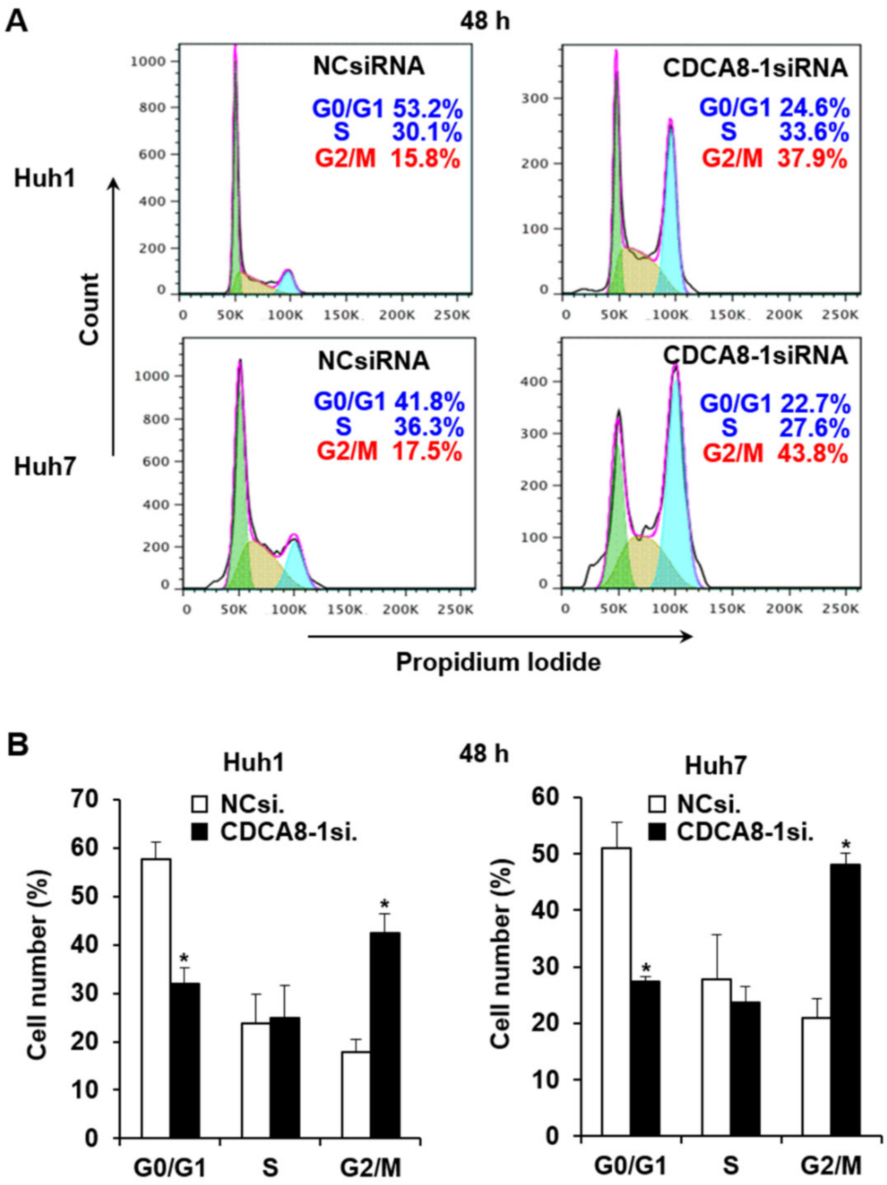

Figure S3. Silencing of CDCA8 leads to G2/M cell cycle arrest in HCC cells. (**A**,**B**) Analysis of cell cycle in HCC cells using flow cytometry. (**A**) At 48 h after siRNA treatment, changes in cell cycle progression were measured and shown in a histogram. (**B**) For each phase of the cell cycle, the percentage of cells is shown in a bar graph. *NCsi.*, negative control siRNA, *CDCA8-1si.*, CDCA8-1siRNA (* $p < 0.05$ by Student's *t*-test with equal variance).

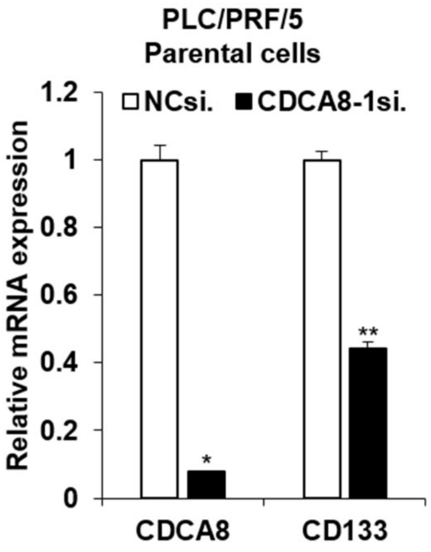

Figure S4. Targeting CDCA8 reduces mRNA expression of CD133 in PLC/PRF/5 parental cell culture. The data are shown relative to GAPDH expression and normalized to NCsiRNA treatment. *NCsi.*, negative control siRNA, *CDCA8-1si.*, CDCA8-1siRNA (* $p < 0.05$, ** $p < 0.01$ by Student's *t*-test).

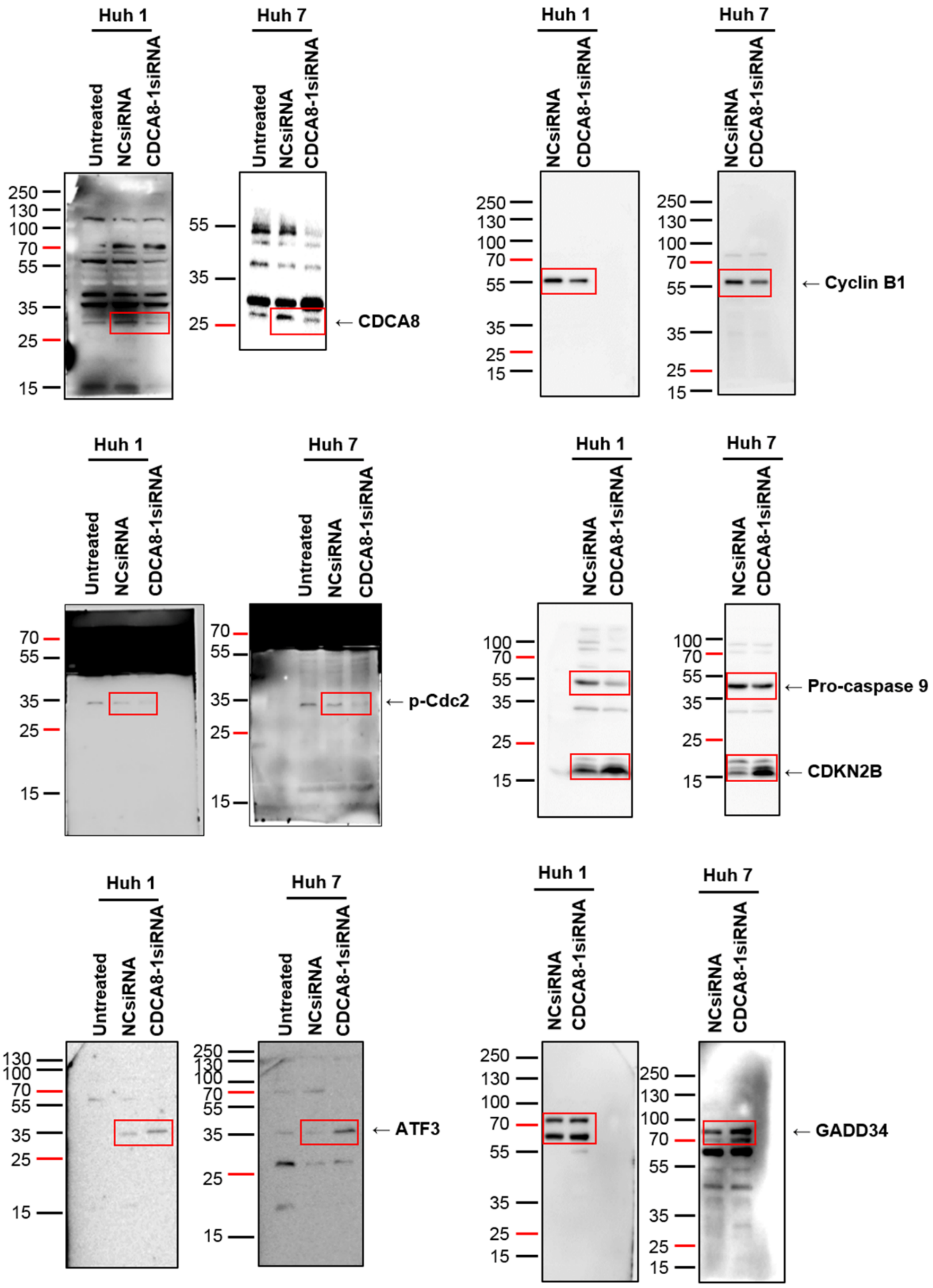

Figure S5. Uncropped original western blots of Figure 6A, which were incubated with CDCA8, cyclin B1, p-cdc2, procaspase-9, CDKN2B, ATF3 or GADD34 antibody The whole images with all the bands and molecular weight markers are shown.

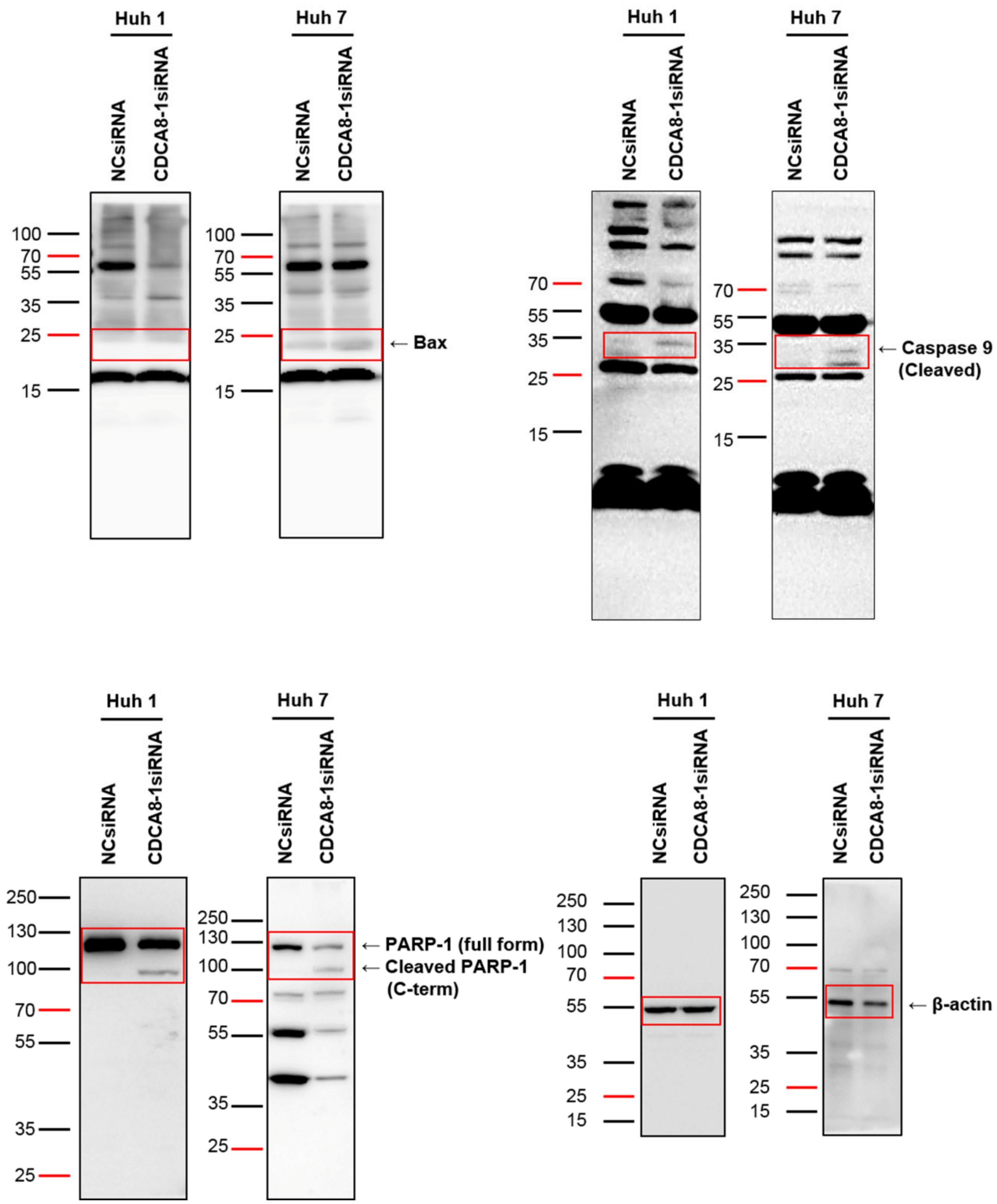

Figure S6. Uncropped original western blots of Figure 6A, which were incubated with Bax, cleaved caspase 9, full form of PARP/cleaved PARP-1 or β-actin antibody. The whole images with all the bands and molecular weight markers are shown.

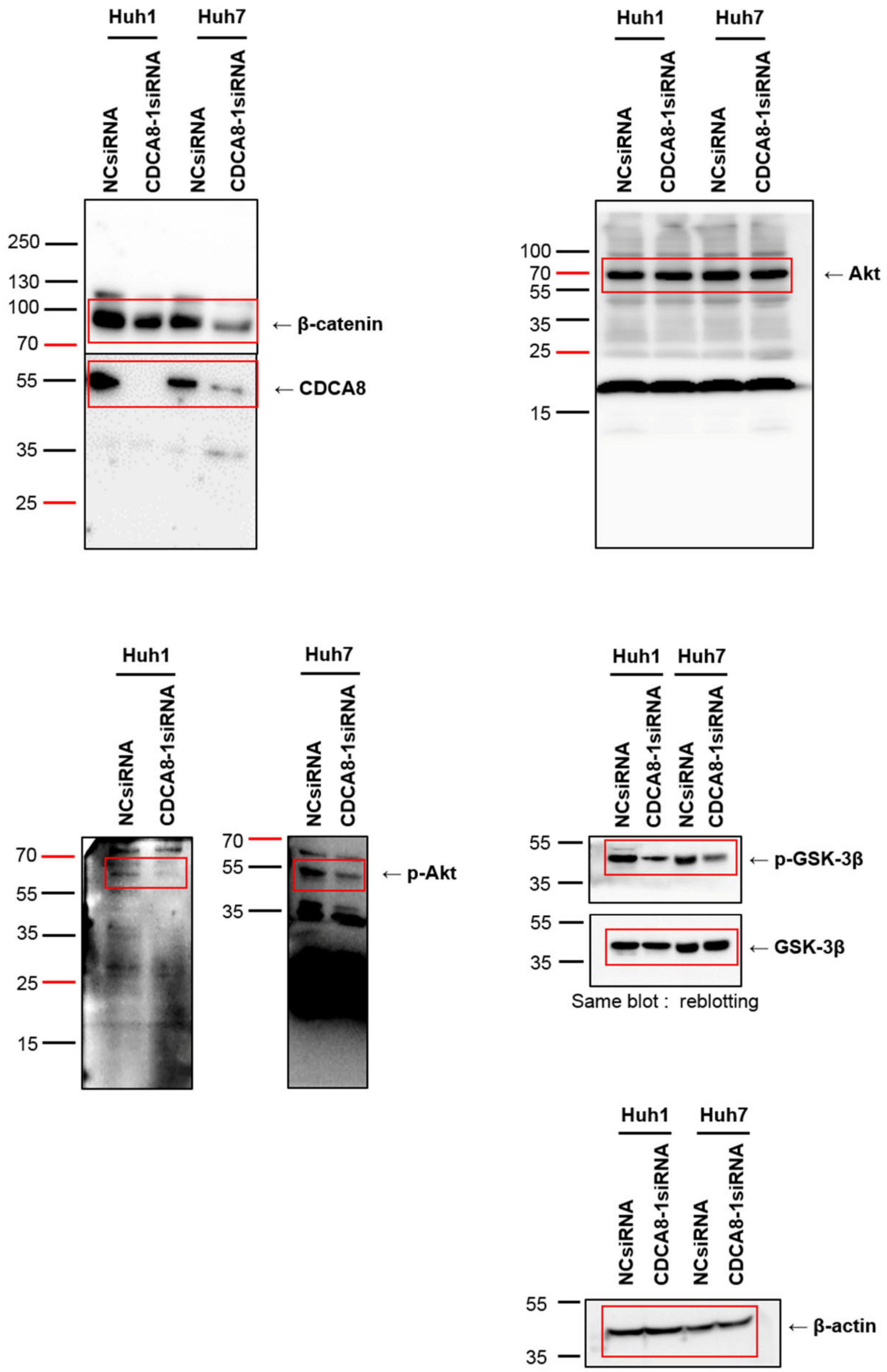

Figure S7. Uncropped original western blots of Figure 7A, which were incubated with CDCA8, β-catenin, Akt, p-Akt, pGSK-3β, GSK-3β or β-actin antibody. The whole images with all the bands and molecular weight markers are shown.

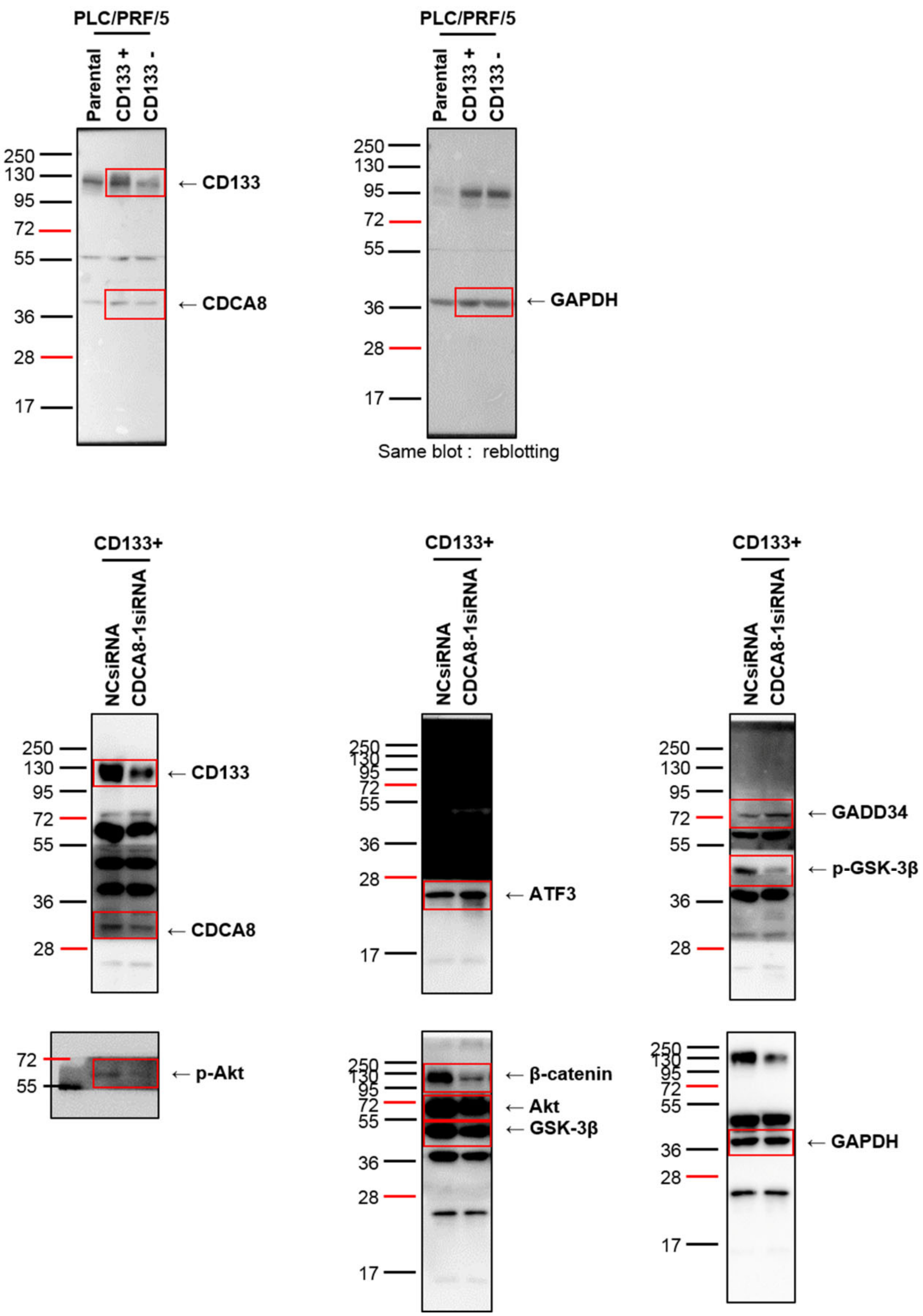

Figure S8. Uncropped original western blots of Figure 8C and 8E, which were incubated with CDCA8, CD133, Akt, p-Akt, β-catenin, pGSK-3β, GSK-3β, ATF, GADD34 or GAPDH antibody The whole images with all the bands and molecular weight markers are shown.

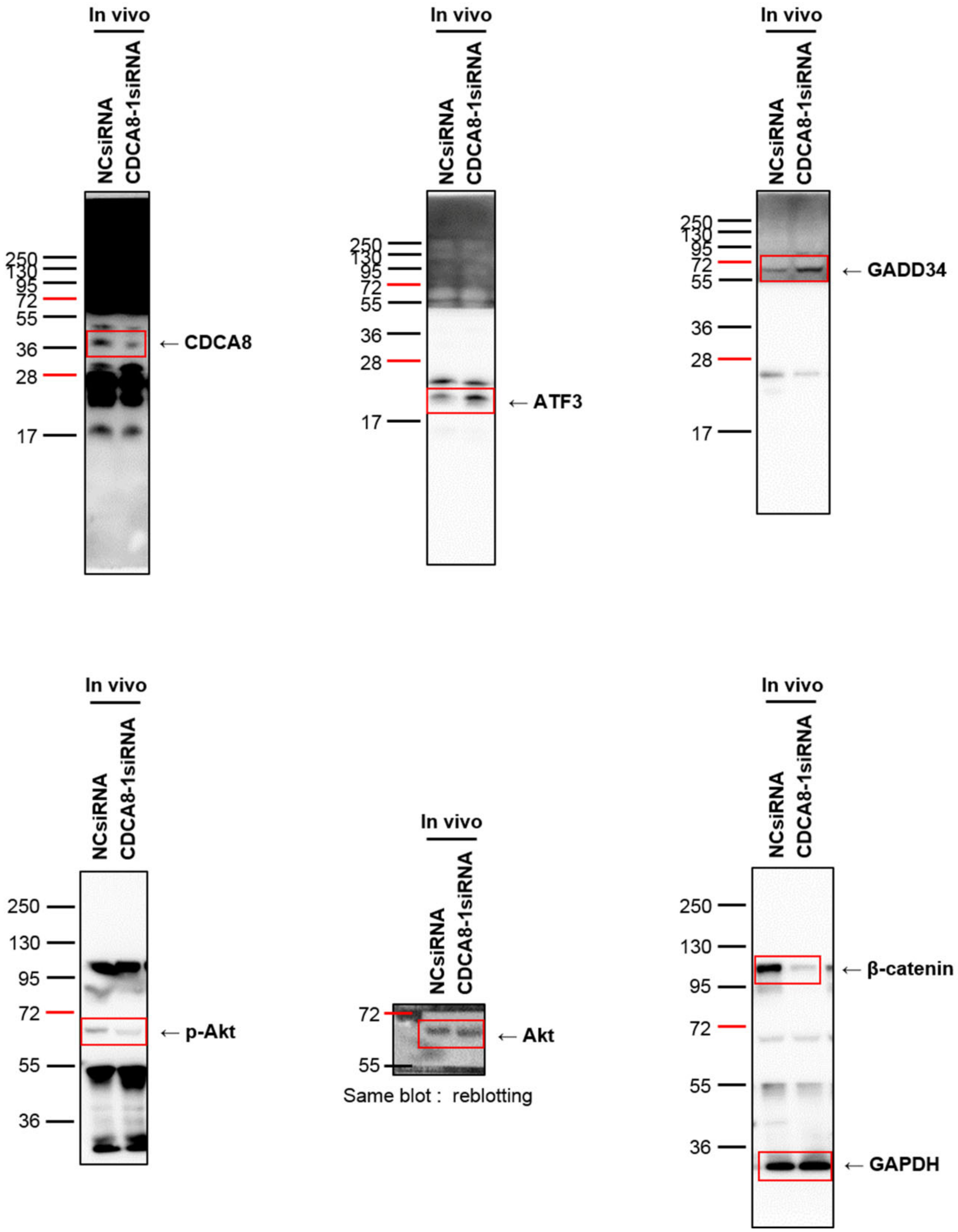

Figure S9. Uncropped original western blots of Figure 9E, which were incubated with CDCA8, ATF3, GADD34, Akt, p-Akt, β-catenin or GAPDH antibody. The whole images with all the bands and molecular weight markers are shown.

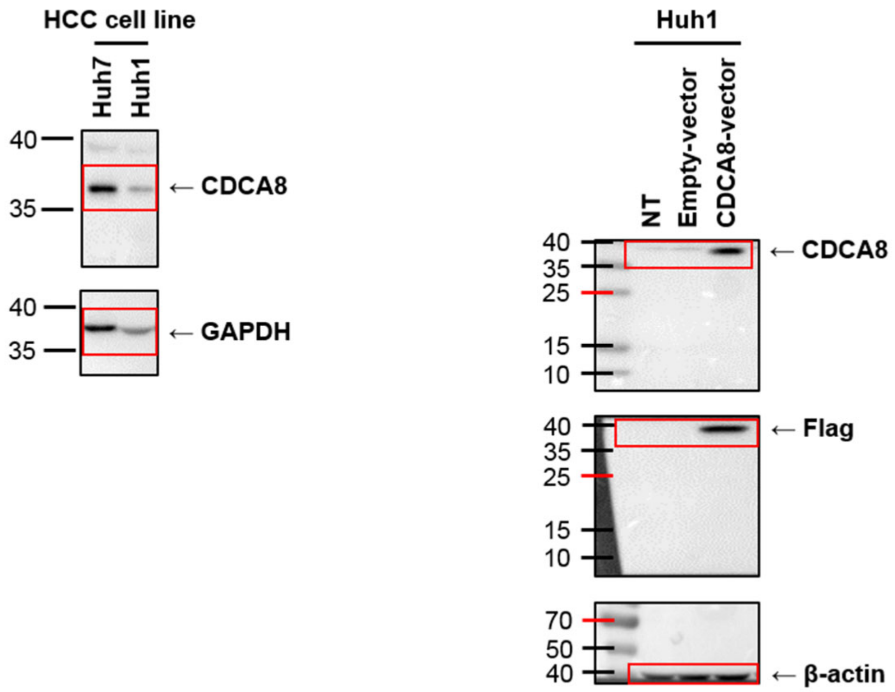

Figure S10. Uncropped original western blots of Figure S2B and S2D, which were incubated with CDCA8, GAPDH, Flag or β-actin antibody. The whole images with all the bands and molecular weight markers are shown.

© 2021 by the authors. Licensee MDPI, Basel, Switzerland. This article is an open access article distributed under the terms and conditions of the Creative Commons Attribution (CC BY) license (http://creativecommons.org/licenses/by/4.0/).
